# Periostin as a Heterofunctional Regulator of Cardiac Development and Disease

**DOI:** 10.2174/138920208786847917

**Published:** 2008-12

**Authors:** Simon J Conway, Jeffery D Molkentin

**Affiliations:** 1 Riley Heart Research Center, Wells Center for Pediatric Research, Indiana University School of Medicine, Indianapolis, IN 46202, USA; 2 Dept. of Pediatrics, University of Cincinnati, Cincinnati Children’s Hospital Medical Center, 3333 Burnet Ave, Cincinnati, OH, 45229, USA

**Keywords:** Periostin, cardiac, signaling, development, hypertrophy, remodeling.

## Abstract

Periostin (Postn) is a heterofunctional secreted extracellular matrix (ECM) protein comprised of four fasciclin domains that promotes cellular adhesion and movement, as well as collagen fibrillogenesis. Postn is expressed in unique growth centers during embryonic development where it facilitates epithelial-mesenchymal transition (EMT) of select cell populations undergoing reorganization. In the heart, Postn is expressed in the developing valves, cardiac fibroblasts and in regions of the outflow track. In the adult, Postn expression is specifically induced in areas of tissue injury or areas with ongoing cellular re-organization. In the adult heart Postn is induced in the ventricles following myocardial infarction, pressure overload stimulation, or generalized cardiomyopathy. Here we will review the functional consequences associated with Postn induction in both the developing and adult heart. The majority of data collected to date suggest a common function for Postn in both development and disease as a potent inducible regulator of cellular reorganization and extracellular matrix homeostasis, although some alternate and controversial functions have also been ascribed to *Postn*, the validity of which will be discussed here.

## INTRODUCTION

In vertebrates four proteins containing fasciclin domains have been described, including Postn, Tgfbi (transforming growth factor-β (TGFβ)-inducible protein [also called βig-H3]), Stabilin-1, and Stabilin-2 [1]. Postn is a 90 kDa secreted protein that contains 4 repetitive fasciclin domains that are similar in sequence to the insect protein fasciclin [2]. Developmentally regulated differential splicing results in both matrix and membrane-associated (*via *amphipathic α-helix) and secreted isoforms. Postn is most closely related to Tgfbi, which like Postn is expressed in collagen-rich connective tissues, remodeling centers and valves, and is induced by TGFβ [3,4]. Fasciclin domain-containing proteins were first functionally characterized in the insect where fasciclin was shown to regulate axon guidance and homophilic cell-cell adhesion [2]. Whilst Postn and Tgfbi each contain 4 fasciclin-like adhesion domains and a single EMI protein-protein interaction module [5], only Tgfbi contains an RGD collagen-association motif [3, 4]. Most fasciclin domain containing proteins are GPI anchored and contain two or four copies of the domain that are thought to mediate cell adhesion through interaction with various integrins. The zebrafish genome also contains z*Postn* and *zTgfbi* homologues, and both zebrafish and mouse *Postn *and* Tgfbi* genes exhibit significant homology to *Drosophila fasciclin-I *[4], suggesting that the vertebrate genes are descendants of a common ancestral gene.

**Fig. (1):**
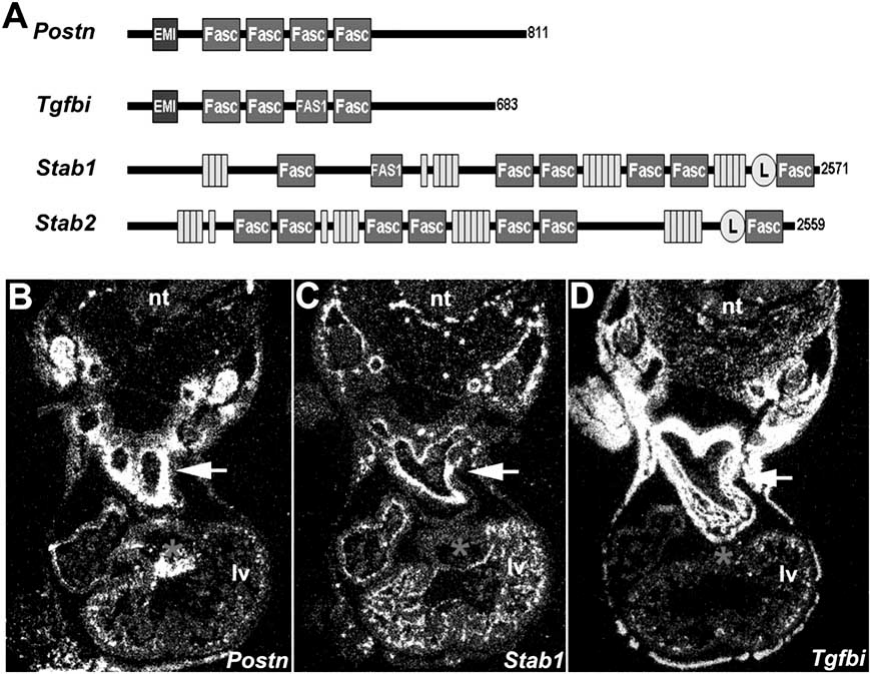
Schematic representation of genes for *Postn, Tgfbi, Stab-1* and *Stab-2* (**A**) Fasciclin (Fasc) domains, the EMI domain, the EGF-like domains (shown as narrow rectangles) and the hyaluronan-binding link domain (shown as circles) are all indicated. The figure is a composite of data obtained using CDART at the NCBI site (http://www.ncbi.nlm.nih.gov/Structure/lexington/lexington.cgi) and PROSITE Database of protein domains, families and functional sites (http://ca.expasy.org/prosite). **(B-D)** Analysis of select fasciclin family members in E10.5 mammalian embryonic hearts. **(B)** *Postn,* **(C)** *Stab-1* and **(D)** *Tgfbi* mRNA expression analysis *via* radioactive *in situ* hybridization (silver grains indicate expression). Both *Postn* and *Tgfbi* are co-expressed in embryonic outflow tract (arrows), cardiac fibroblasts and the pericardium. However, *Postn* outflow tract expression is confined to the endocardial cushions, whilst *Tgfbi* is expressed in both outflow tract, endocardial cushions and adjacent myocardial cuff. Additionally, only *Postn* is robustly expressed in atrio-ventricular cushions (indicated by *). In contrast, the transmembrane *Stab-1* gene is expressed in adjacent endothelial lineages **(C)** *Stab-2* expression is not shown as it is confined to embryonic liver and is absent from the embryonic heart.^4^

In contrast to the secreted Postn and Tgfbi proteins, the high molecular mass Stabilin-1 (Stab-1) and Stabilin-2 (Stab-2) proteins uniquely contain 7 fasciclin adhesion domains, numerous epidermal growth factor (EGF)-like domains, a link hyaluronan (HA)-binding domain, and a predicted transmembrane domain (Fig. **1A**). While *Stab-1* and *Stab-2* are structurally similar, they appear to have unique functions as Stab-1 acts to clear unwanted self-molecules, while Stab-2 acts as a scavenger receptor for HA and AGE-modified proteins [6,7]. Both putative Stab-1 and Stab-2 receptors are expressed during development and within endothelial tissues and alternatively activated macrophages in the adult spleen, liver, lymph nodes and placenta. All four mammalian fasciclin domain-containing genes are expressed in the adult heart, whilst only *Postn, Tgfbi* and* Stab-1* are present in the *in utero* developing heart (Fig. **1B**-**D**) [4]. Even though several independent but complimentary *Postn* mice knockout studies have begun to shed light upon the requirement and possible function of Postn within the cardiovascular system, the roles of *Tgfbi, Stab-1* and S*tab-2* during cardiovascular development and heart homeostasis are presently unknown. 

As a secreted ECM protein that associates with areas of fibrosis, Postn can directly interact with other ECM proteins such as fibronectin, tenascin-C, collagen I, collagen V, and heparin [8-10].  Postn can serve as a ligand for select integrins, such as α_v_β_3_, α_v_β_5_, and α_4_β_6_, where it can affect the ability of cells (fibroblasts or cancer cells) to migrate and/or undergo a EMT in select tissues during pathological disease progression [11,12]. However, it remains unclear whether this ligand-receptor association also occurs during normal homeostasis. Postn up regulation is also involved in cell survival and angiogenesis, and has become known as a promising marker for tumor progression in various types of human cancers [13-15]. 

## *Postn* EXPRESSION DURING HEART DEVELOPMENT

Takeshita *et al.* originally cloned mouse *Postn*, which they designated *osteoblast-specific factor-2 (OSF-2)*, as it was robustly expressed within the osteoblastic MC3T3-E1 cells and bone samples [16]. Mouse and human Postn share 89.2% amino acid identity, suggesting a high degree of functional conservation. *Postn* shows a dynamic expression profile, both developmentally and in adult tissues that are undergoing remodeling or active stress. *Postn* is expressed in the developing endocardial cushions of the heart and the mature valves, the periosteum and periodontal ligament, injured vessels, tumors and metastatic cancer cells, and in cells undergoing EMT [11, 17-19]. With respect to cell type of expression, Postn appears to be expressed exclusively in endocardial cushion and fibroblast lineages, or in cells that adopt fibroblast-like characteristics following an injury event [8, 11, 20-23]. In addition to fibroblasts, Postn is expressed in other structures within the developing heart that may or may not be fibroblast in origin such as the valvular attachment apparatus, chordae tendineae and epicardial/pericardial structures, but is absent from the cardiomyocyte lineage itself [4, 10, 16, 17, 20, 21]. 

## DEVELOPMENTAL DEFECTS ASSOCIATED WITH *Postn* DELETION IN MICE

To investigate the potential developmental functions of Postn, a gene deletion strategy was undertaken in mice in which the first exon was replaced with a *lacZ* reporter gene [24] or exons 4 through 10 encoding three of the 4 fasciclin domains were deleted by insertion of a neomycin cassette [21]. Both mutants produced null alleles, and will hereafter be termed *Postn^lacZ/lacZ ^*and *Postn*^-/-^ mice. Although *Postn* is widely expressed in many developing organ systems, the majority of both *Postn^lacZ/lacZ ^*and *Postn*^-/-^ mice survived well into adulthood, although they presented with smaller overall body weights. Even though ~14% of the *Postn^lacZ/lacZ ^*nulls die before weaning, the surviving *Postn^lacZ/lacZ ^*mice develop an early-onset periodontal ligament (PDL) phenotype, with profound ultrastructural and histological changes in ameloblasts and enamel defects. *Postn^lacZ/lacZ ^*mutants up regulate collagen III and exhibit a disorganized ameloblast layer that secretes an amorphous hypermineralized matrix. Significantly, placing *Postn^lacZ/lacZ ^*mice on a soft diet reduced growth retardation, improved fertility and alleviated mechanical strain on the PDL but not postnatal lethality [24]. Subsequent deletion of *Postn *by another group also revealed a similar phenotype of smaller body size, as well as defective tooth development [9]. Ensuing analysis indicated *Postn^lacZ/lacZ ^*mice exhibited severe periodontal defects after tooth eruption [25]. Interestingly, tooth clipping to remove masticatory forces rescued the periodontal defects. Using Flexicell culture apparatus to generate mechanical stress, *Postn *expression increased in strained PDL cells. This was preceded by a transient increase in *Tgfβ* mRNA, and *Postn* elevation was blocked by addition of TGFβ-neutralizing antibodies [25]. Combined, these data suggest that *Postn *is essential for the proper function of the PDL during absorbance of strain and maintenance of the integrity in response to mechanical stresses. 

## CARDIOVASCULAR PHENOTYPE OF MICE LACKING *Postn*

Analysis of the cardiovascular system in *Postn^lacZ/lacZ^* and *Postn*^-/-^ mice revealed neonatal valvular-dependent lethality in a minority of offspring, and latent disease with leaflet abnormalities in the viable majority of offspring [20, 26]. Surviving null leaflets are truncated, containing ectopic cardiomyocytes and smooth muscle, mis-expression of the cartilage proteoglycan aggrecan with disorganized matrix stratification. This phenotype is consistent with the islands/pockets of cartilage reported in adult valves including mice and human [20, 27, 28], and with the phenotype of *Smad6^-/-^* in which cartilage, bone, bone marrow, and even blood cells were formed within both AV and aortic valve leaflets [29]. Collectively, these findings suggest cushion cells are multipotential cells whose differentiation potential is normally restricted to primarily a fibroblastic lineage. Assessment of collagen production, 3D-lattice formation ability, and $\text{TGF}\tilde{\beta }$-responsiveness indicate that *Postn*-deficient fibroblasts are unable to support normal valvular remodeling [20]. Furthermore, null fibroblasts exhibit reduced ability to reorganize and contract 3D-collagen lattices, and a diminished response to increasing TGFβ concentrations. In fact, Postn can directly bind collagen type-I [10]. These observations collectively suggest that loss of *Postn* results in inappropriate differentiation of mesenchymal cushions and valvular abnormalities *via *a TGFβ-dependent pathway during establishment of mature valve leaflets. 

*Alk3* (type IA receptor for bone morphogenic proteins (BMPs)) has been shown to be required for *Postn* expression in remodeling AV valves [30]. *Postn* was initially normally expressed in *cGata6-Cre/Alk3* mutant E11.5 cushions but was appreciably reduced in mutant E14.5 atrioventricular (AV) leaflets. Given that TGFβ2 expression is also decreased in *Alk3*^–/– ^mutants [31], the cardiac cushions of mutant mice may therefore be hypoplastic secondary to decreased TGFβ2–mediated endocardial cushion EMT. In support of this, exogenous chick BMP2 or constitutively active *Alk6* (type-IB BMP receptor) retrovirus significantly promoted expression of *Postn* in AV cushion explant assays [32]. As BMP2 has also been shown to induce TGFβ2 in this culture system [33] and BMP2 is known to function synergistically with TGFβ3 in the regulation of endocardial cushion EMT [34], BMPs may induce Postn by activating an endocardial TGFβ autocrine loop. In explanted diseased pediatric cardiac tissues from stenotic bicuspid aortic valves, Postn expression is largely absent from regions where ECM trilaminar stratification was lost, collagen fibrils disorganized and elastin fiber content reduced [20]. Combined with the mouse genetic disruption data, these results suggest that Postn may be required *in utero* for events that manifest themselves in postnatal and adult life. Specifically, Postn plays multiple roles as a primary responder molecule and may be linked to ECM deposition/re-organization during maturation and homeostasis of fibrous valve leaflets and maintenance of PDL integrity in response to mechanical stresses. 

Connective tissue disorders are among the most common congenital defects in humans and constitute numerous adult diseases. Cardiac valve thickening and aortic dilation are major cardiovascular phenotypes of Marfans, and Marfans-like disorders, and are manifested through an alteration in collagen fibers and microfibril assembly [35]. Marfans patients have increased Postn protein as well as increased TGFβ and BMP signaling in their thickened heart valves, which suggests that Postn is positively regulated by TGFβ and BMP [36, 37]. Despite the complex and intriguing correlation of deregulated *Postn* expression levels in both normal and pathological transformation conditions, very little is known about how *Postn* is transcriptionally controlled. Thus, unraveling the molecular mechanisms that regulate *Postn* expression could prove insightful. For example, during osteoblast differentiation, transcription of *Postn* may be regulated by the bHLH transcription factor *Twist*, which itself is associated with EMT during tumor progression [38, 39]. A 3.9kb *Postn* proximal promoter region was isolated and shown to drive tissue-specific expression in the neural crest-derived Schwann cell lineage and in a subpopulation of *Postn*-expressing cells in the cardiac outflow tract and endocardial cushions [4]. Further analysis of the *Postn* promoter also implicated the ubiquitous transcription factor *Ying Yang-1* (*YY1*) in regulating expression, which is interesting given YY1’s role as an initiator of tumorigenesis and inhibitor of important cell-cycle progression genes [40-42]. Thus, a better understanding of *Postn* transcriptional regulatory mechanisms might also suggest additional molecular pathways that underlie its developmental functions. 

## ROLE OF *Postn* IN ADULT HEART DISEASE

**Fig. (2):**
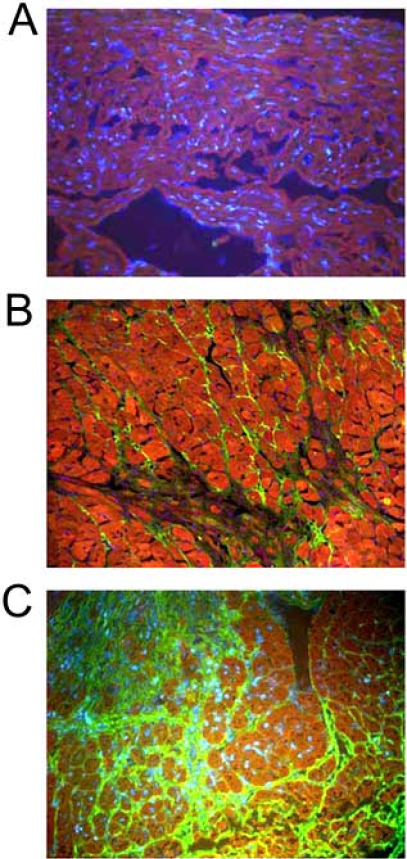
Distribution of endogenous Postn protein expression in adult mouse hearts at base line **(A)**, subjected to pressure overload for 8 weeks **(B)**, or 2 weeks after myocardial infarction **(C)**. A Postn specific antibody was used (green fluorescence) and a myocyte-specific contractile protein (red fluorescence). Panels B and C show abundant Postn protein accumulation in the extracellular space from the injury stimulus.

It is likely that the developmental functions of *Postn* discussed above in facilitating proper organization of the ECM and in affecting cellular trafficking in remodeling hotspots is selectively re-employed in the adult organism. For example, Postn is expressed in collagen-rich connective tissues, remodeling centers, at sites of injury and inflammation, and is similarly induced by TGF-β [9, 13, 19-21, 43-45]. Specifically, myocardial infarction (MI) or pressure overload stimulation to the adult heart induces abundant re-expression of Postn from resident fibroblasts located between myocytes within the heart parachyma proper (Fig. **2A**-**C**), where prior to such stimulation the major expression of Postn in the heart is within the collagen rich environment of the valves [20, 21, 45-48]. Postn expression is also induced at sites of vascular injury and hyperplasia [18, 49, 50], in the lung after injury/fibrosis [8, 51], in and around tumors [13], and at wound sites [52]. While not unequivocally proven, these various studies suggest a common biologic response whereby *Postn* becomes re-expressed at injury or inflammatory sites within the adult organism, often associated with a need for ECM and cellular migration, not unlike many developmental processes associated with Postn expression.

The heart contains a fibrosis skeleton of collagen and other ECM components that maintains overall ventricular geometries and the proper organization of myocytes within aligned strips that wrap around the heart for optimal force generation [53]. Remodeling of the fibrous skeleton and the ECM surrounding individual myocytes is critically involved in hypertrophic and dilatory growth of the heart in response to either pathologic or physiologic stimulation [53]. Long-standing pressure overload associated with hypertension, viral myocarditis, postpartum cardiomyopathy, and hypertrophic cardiomyopathy all induce significant alterations in the ECM and can lead to fibrotic heart disease and loss of ventricular compliance [54].MI associated with occlusion of a major coronary artery leads to rapid cell death of an entire region of the ventricular wall or septum, resulting in an inflammatory response and subsequent formation of a fibrous scar that is rich in collagen but lacking in contractile capacity [55]. As discussed above, Postn is minimally expressed in the adult ventricles unless a pathologic stress or injury occurs. The function of Postn upon re-expression in the injured myocardium is currently an area of controversy given disparate accounts in the literature, although most reports suggest a function that is tied to the ECM, fibrotic remodeling, and the movement of cells through an injured or reorganizing area. 

We reported that *Postn^-/-^* mice had an altered fibrotic response and were unable to form proper scars after MI injury, leading to greater rates of wall rupture and death [21], a result that was independently confirmed the following year in separately generated *Postn* null mice [45]. Conversely, we generated transgenic mice over expressing full-length *Postn* in the heart, which protected from wall rupture following MI injury [21]. Abundant Postn over expression in the heart did not induce fibrosis, although it did eventually promote a mild hypertrophy response that we interpret to be associated with integrin signaling (see below). Another interesting aspect of *Postn^-/-^* mice is that they were less susceptible to fibrotic heart disease associated with long-term pressure overload or MI, resulting in better ventricular performance [21]. These results suggest that loss of *Postn* is actually protective to the heart long-term, provided the initial insult is survived during the scar formation period if the insult was an MI (1-7 days). 

Three other groups have also attempted to alter expression of Postn or an alternately spliced isoform of Postn in the adult heart to examine its role in cardiac adaptation or maladaptation [23, 56, 57]. Katsuragi *et al*. attempted to augment or inhibit Postn expression in the adult rat heart using a plasmid DNA transfection protocol or a one-time infusion of antisense oligonucleotides, respectively [23]. Katsuragi *et al.* contended that using this method to over express Postn caused ventricular dilation and reduced cardiac function, although Postn protein expression was not examined, rendering these results difficult to interpret. This pathologic phenotype associated with Postn plasmid DNA transfection is also in stark contrast to data obtained in Postn over expressing transgenic mice, which showed no such effect, even at high levels of sustained expression for up to 12 months [21]. In another study, Litvin *et al*. injected an adenovirus encoding an alternatively spliced isoform of Postn (called PLF) into the heart, which after 7 days caused a minor increase in cardiac hypertrophy [56]. These results are consistent with Postn transgenic mice [21], although the adenoviral approach employed by Litvin *et al*. may have hastened the hypertrophic response due to the adenoviral infection itself. Thus, over expression of Postn is relatively innocuous to the adult heart, although it does eliminate rupture susceptibility following MI and it can produce a mild hypertrophy response with aging.

Results reported from a third group are perhaps the most controversial, as they suggest an entirely novel function for *Postn* in mediating cardiomyocyte proliferation and the subsequent repair of injured myocardium [57]. In addition to reporting increased proliferation, Kühn *et al.* claimed that Postn prevented fibrosis and reduced cardiac hypertrophy after an injury event, results that are not consistent with Oka *et al. *[21]. A potential reason for these differences may be that Kühn *et al*. utilized a recombinant truncated form of Postn (amino acids 22-669 of 811) in ascribing biological effects [57]. Using this partial protein, as well as a full-length protein, the authors reported increased proliferation rates in adult rat cardiac myocytes cultured for 9 days [57]. These later results may also be compromised given the known process of de-differentiation that occurs in adult myocytes cultured for this length of time, possibly rendering the cells artificially permissive to proliferation. More remarkably, Kühn *et al.* reported that application of the truncated form of Postn with a gelfoam patch onto infarcted hearts reduced fibrosis, reduced cardiac hypertrophy, increased myocyte proliferation, and improved cardiac function [57]. However, over expression of full-length *Postn* in the mouse heart by transgenesis (from the cardiomyocytes-restricted α-myosin heavy chain promoter), which results in the proper accumulation of Postn protein in the ECM without detectable intracellular retention, did not increase myocyte number at baseline, nor did it augment indices associated with cardiac repair after MI injury [21]. Moreover, Oka *et al.* reported that *Postn^-/-^* mice had reduced fibrosis and improved function after MI, provided they did not succumb to wall rupture, and *Post*n over expressing transgenic mice actually developed cardiac hypertrophy and were not protected from negative remodeling and fibrosis after MI [21]. Thus, the *in vivo* results of Kühn *et al.* are essentially opposite of Oka *et al. *While multiple reasons may underlie these differences, Oka *et al.* relied on genetically modified mice to alter Postn expression compared with non-genetic approaches by Kühn *et al.* in which recombinant and truncated Postn was employed in conjunction with a questionable control group. For example, the control group for the MI results of Kühn *et al.* simply relied on buffer treatment [57]. A more reliable control would have been the use of gelfoam patches loaded with a non-specific recombinant protein generated in *E. coli* with the same potential for LPS contamination or the effects of the gelfoam patch itself. Thus, the contention that Postn promotes physiologically meaningful cardiac myocyte proliferation in the heart following MI injury is far from established. We favor the alternate hypothesis that Postn mostly functions in collagen fibrillogenesis and cellular remodeling.

## *Postn* SIGNALING MECHANISMS IN THE ADULT HEART

Alterations in cardiac remodeling and hypertrophy at the hands of Postn might occur by two different, but potentially overlapping mechanisms. Postn might simply regulate the integrity of the ECM through its ability to bind multiple ECM proteins, such as tenascin-C, fibronectin, collagen V, collagen I, aggrecan and heparin, thus affecting the structural integrity of the adult heart matrix or stretch-sensitive signaling [8, 10, 58]. Indeed, collagen fibrils from Postn^-/-^ mice were reduced in size, somewhat disorganized, and less efficiently cross-linked [10, 45]. The *Postn^lacZ/lacZ^* PDL ECM is disorganized and exhibits a reduced ability to absorb masticatory mechanical stresses [24]. Similarly, skin from *Postn^-/-^* mice has lower tensile strength and a difference in elasticity [10]. Hence, Postn may secondarily impact the structural properties of the heart by altering the composition and performance of the ECM. Such alterations in the ECM could easily affect cardiac remodeling by simply modifying the strength and stretch characteristics of the tissue with a commensurate impact on valvular closing and myocyte signal transduction. Another related issue is that Postn appears to be a more pliable substrate compared with fibronectin and vitronectin, likely affecting how mesenchymal cells (fibroblasts, inflammatory cells, stem cells) migrate within the heart following injury [11]. Indeed, work in the cancer field has shown that transformed cells preferentially secrete Postn to facilitate invasiveness and metastatic activity [11, 12, 59]. This same static feature of ECM pliability may also underlie invasiveness of mesenchymal cells into endocardial cushions in the presence of Postn in the developing heart valves [19]. 

A second potential mechanism whereby Postn affects the phenotype of the adult heart is by attachment-dependent signaling through cell adhesion molecules. Postn is an integrin binding protein and its attachment with fibroblasts or myocytes may impart a signal that affects how these cells perceive their environment and respond to stress or other remodeling signals. As mentioned earlier, Postn supports α_v_β_3_, α_v_β_5_, and α_4_β_6_, integrin attachment/signaling to facilitate EMT and metastatic activity [11, 59], as well as movement of cells within the developing bone centers and PDLs [11, 22, 9, 12, 24]. This integrin dependent interaction could impart a direct signal to cardiac myocytes that affects their hypertrophic growth or the milieu of other cells that surround myocytes and their paracrine signals. Alternatively, Postn might serve as a unique substrate for various inflammatory, mesenchymal, and stem cells to signal the need for repair in a given region of the myocardium based on unique integrin attachment properties. Finally, Postn might also signal through a homotypic interaction with the membrane bound fasciclin domain containing receptors Stab-1 or Stab-2 found on inflammatory or mesenchymal cells. Indeed, Stab-1 is highly expressed on macrophages and adjacent endothelial cells where it might be attracted to or modified within the injury area through a homotypic interaction with Postn and/or Tgfbi [4, 60, 61]. 

**Fig. (3):**
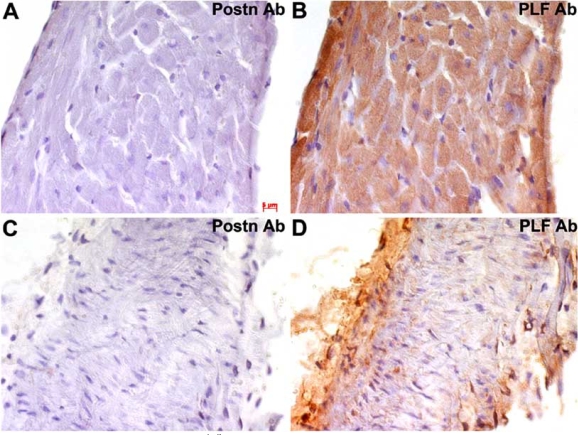
Comparison of Postn and PLF within *Postn^lacZ^* nulls using antibodies directed against exon 21 (Postn-specific antibody) and *Postn* exon 17 (for PLF antibody). Brown staining shows positive immunohistochemistry for Postn or PLF. The results show that Postn is absent in adult cardiac myocytes **(A)** and null aorta **(C)**, but that PLF antibody reactivity is detectable within *Postn^lacZ/lacZ^* knockout cardiac myocytes **(B)** and null aorta **(D)**. These results suggest that the PLF antibody used by Litvin *et al.* [56] to claim myocyte-specific expression of PLF is likely due to one or more other non-specific proteins recognized by the PLF antibody or an unknown cryptic start site within the *Postn* C-terminus.

It has also been reported that a “*periostin-like factor*” (PLF) may be over expressed in failing human and rat cardiomyocytes and is localized to the cardiac myocyte itself [56]. However, PLF is actually one of four Postn isoforms expressed in the heart, and all four isoforms are missing in *Postn^lacZ/lacZ^* mice using a highly characterized antibody (Fig. **3A**-**D**) [62]. This differentially spliced non-secreted isoform was originally identified by Kudo *et al*. [63]. Thus it is unclear why this particular Postn isoform was renamed PLF. Despite these issues, none of the alternately spliced isoforms off Postn are expressed in myocytes, and all appear to be exclusively made in fibroblasts from within the heart [10, 20, 21].

## References

[1] Thapa N, Lee BH, Kim IS (2007). TGFBIp/betaig-h3 protein: a versatile matrix molecule induced by TGF-beta. Int. J. Biochem. Cell Biol.

[2] Bastiani MJ, Harrelson AL, Snow PM, Goodman CS (1987). Expression of fasciclin I and II glycoproteins on subsets of axon pathways during neuronal development in the grasshopper. Cell.

[3] LeBaron RG, Bezverkov KI, Zimber MP, Pavelec R, Skonier J, Purchio AF (1995). Beta IG-H3, a novel secretory protein inducible by transforming growth factor-beta, is present in normal skin and promotes the adhesion and spreading of dermal fibroblasts *in vitro*. J. Invest. Dermatol.

[4] Lindsley A, Li W, Wang J, Maeda N, Rogers R, Conway SJ (2005). Comparison of the four mouse fasciclin-containing genes expression patterns during valvuloseptal morphogenesis. Gene Expr. Patterns.

[5] Callebaut I, Mignotte V, Souchet M, Mornon JP (2003). EMI domains are widespread and reveal the probable orthologs of the Caenorhabditis elegans CED-1 protein. Biochem. Biophys. Res. Commun.

[6] Politz O, Gratchev A, McCourt PAG, Schledzewski K, Guillot P, Johansson S, Svineng G, Franke P, Kannicht C, Kzhyshkowska J, Longati P, Velten FW, Johansson S, Goerdt S (2002). Stabilin-1 and -2 constitute a novel family of fasciclin-like hyaluronan receptor homologues. Biochem. J.

[7] Kzhyshkowska J, Gratchev A, Goerdt S (2006). Stabilin-1, a homeostatic scavenger receptor with multiple functions. J. Cell Mol. Med.

[8] Takayama G, Arima K, Kanaji T, Toda S, Tanaka H, Shoji S, McKenzie AN, Nagai H, Hotokebuchi T, Izuhara K (2006). Periostin: a novel component of subepithelial fibrosis of bronchial asthma downstream of IL-4 and IL-13 signals. J. Allergy Clin. Immunol.

[9] Kii I, Amizuka N, Minqi L, Kitajima S, Saga Y, Kudo A (2006). Periostin is an extracellular matrix protein required for eruption of incisors in mice. Biochem. Biophys. Res. Commun.

[10] Norris R, Damon B, Mironov V, Kasyanov V, Ramamurthi A, Moreno-Rodriguez R, Trusk T, Potss J, Goodwin RL, Davis J, Hoffman S, Wen X, Sugi Y, Kern CB, Mjaatvedt CH, Turner D, Oka T, Conway SJ, Molkentin JD, Forgacs G, Markwald R (2007). Periostin regulates collagen fibrillogenesis and the biomechanical properties of connective tissues. J. Cell Biochem.

[11] Gillan L, Matei D, Fishman DA, Gerbin CS, Karlan BY, Chang DD (2002). Periostin secreted by epithelial ovarian carcinoma is a ligand for alpha(V)beta(3) and alpha(V)beta(5) integrins and promotes cell motility. Cancer Res.

[12] Baril P, Gangeswaran R, Mahon PC, Caulee K, Kocher HM, Harada T, Zhu M, Kalthoff H, Crnogorac-Jurcevic T, Lemoine NR (2007). Periostin promotes invasiveness and resistance of pancreatic cancer cells to hypoxia-induced cell death: role of the beta(4) integrin and the PI3k pathway. Oncogene.

[13] Kudo Y, Siriwardena BS, Hatano H, Ogawa I, Takata T (2007). Periostin: novel diagnostic and therapeutic target for cancer. Histol. Histopathol.

[14] Tilman G, Mattiussi M, Brasseur F, van Baren N, Decottignies A (2007). Human periostin gene expression in normal tissues, tumors and melanoma: evidences for periostin production by both stromal and melanoma cells. Mol. Cancer.

[15] Bao S, Ouyang G, Bai X, Huang Z, Ma C, Liu M, Shao R, Anderson RM, Rich JN, Wang XF (2004). Periostin potently promotes metastatic growth of colon cancer by augmenting cell survival *via* the Akt/PKB pathway. Cancer Cell.

[16] Takeshita S, Kikuno R, Tezuka K, Amann E (1993). Osteoblast-specific factor 2: cloning of a putative bone adhesion protein with homology with the insect protein fasciclin 1. Biochem. J.

[17] Kruzynska-Frejtag A, Machnicki M, Rogers R, Markwald R, Conway SJ (2001). Periostin (an osteoblast-specific factor) is expressed within the embryonic mouse heart during valve formation. Mech. Dev.

[18] Li G, Oparil S, Sanders JM, Zhang L, Dai M, Chen LB, Conway SJ, McNamara CA, Sarembock IJ (2006). Phosphatidylinositol-3-kinase signaling mediates vascular smooth muscle cell expression of periostin *in vivo* and *in vitro*. Atherosclerosis.

[19] Butcher JT, Norris RA, Hoffman S, Mjaatvedt CH, Markwald R (2007). Periostin promotes atrioventricular mesenchyme matrix invasion and remodeling mediated by integrin signaling through Rho/PI 3-kinase. Dev. Biol.

[20] Snider P, Hinton RB, Moreno-Rodriguez RA, Wang J, Rogers R, Lindsley A, Li F, Ingram DA, Menick D, Field L, Firulli AB, Molkentin JD, Markwald R, Conway SJ (2008). Periostin Is Required for Maturation and Extracellular Matrix Stabilization of Noncardiomyocyte Lineages of the Heart. Circ. Res.

[21] Oka T, Xu J, Kaiser RA, Melendez J, Hambleton M, Sargent MA, Lorts A, Brunskill EW, Dorn GW 2nd, Conway SJ, Aronow BJ, Robbins J, Molkentin JD (2007). Genetic manipulation of periostin expression reveals a role in cardiac hypertrophy and ventricular remodeling. Circ. Res.

[22] Wilde J, Yokozeki M, Terai K, Kudo A, Moriyama K (2003). The divergent expression of periostin mRNA in the periodontal ligament during experimental tooth movement. Cell Tissue Res.

[23] Katsuragi N, Morishita R, Nakamura N, Ochiai T, Taniyama Y, Hasegawa Y, Kawashima K, Kaneda Y, Ogihara T, Sugimura K (2004). Periostin as a novel factor responsible for ventricular dilation. Circulation.

[24] Rios H, Koushik SV, Wang H, Wang J, Zhou HM, Lindsley A, Rogers R, Chen Z, Maeda M, Kruzynska-Frejtag A, Feng JQ, Conway SJ (2005). Periostin null mice exhibit dwarfism, incisor enamel defects, and an early-onset periodontal disease-like phenotype. Mol. Cell Biol.

[25] Rios H, Ma D, Xie Y, Giannobile W, Bonewald LF, Con-way SJ, Feng J (2008). Periostin is essential for the integrity and function of the periodontal ligament during occlusal loading. J. Periodont.

[26] Norris RA, Moreno-Rodriguez R, Sugi Y, Hoffman S, Amos J, Hart MM, Potts JD, Goodwin RL, Markwald R (2008). Periostin regulates atrioventricular valve maturation. Dev. Biol.

[27] Icardo JM, Colvee E (1995). Atrioventricular valves of the mouse: III Collagenous skeleton and myotendinous junction. Anat. Rec.

[28] Mulholland DL, Gotlieb AI (1996). Cell biology of valvular interstitial cells. Can. J. Cardiol.

[29] Galvin KM, Donovan MJ, Lynch CA, Meyer RI, Paul RJ, Lorenz JN, Fairchild-Huntress V, Dixon KL, Dunmore JH, Gimbrone MA Jr, Falb D, Huszar D (2000). A role for smad6 in development and homeostasis of the cardiovascular system. Nat. Genet.

[30] Gaussin V, Morley GE, Cox L, Zwijsen A, Vance KM, Emile L, Tian Y, Liu J, Hong C, Myers D, Conway SJ, Depre C, Mishina Y, Behringer RR, Hanks MC, Schneider MD, Huylebroeck D, Fishman GI, Burch JB, Vatner SF (2005). Alk3/Bmpr1a receptor is required for development of the atrioventricular canal into valves and annulus fibrosus. Circ. Res.

[31] Gaussin V, Van de PT, Mishina Y, Hanks MC, Zwijsen A, Huylebroeck D, Behringer RR, Schneider MD (2002). Endocardial cushion and myocardial defects after cardiac myocyte-specific conditional deletion of the bone morphogenetic protein receptor ALK3. Proc. Natl. Acad. Sci. USA.

[32] Inai K, Norris RA, Hoffman S, Markwald RR, Sugi Y (2008). BMP-2 induces cell migration and periostin expression during atrioventricular valvulogenesis. Dev. Biol.

[33] Sugi Y, Yamamura H, Okagawa H, Markwald R (2004). Bone morphogenetic protein-2 can mediate myocardial regulation of atrioventricular cushion mesenchymal cell formation in mice. Dev. Biol.

[34] Yamagishi T, Nakajima Y, Miyazono K, Nakamura H (1999). Bone morphogenetic protein-2 acts synergistically with transforming growth factor-beta3 during endothelial-mesenchymal transformation in the developing chick heart. J. Cell Physiol.

[35] Ramirez F, Saka  LY, Rifkin DB, Dietz HC (2007). Extracellular microfibrils in development and disease. Cell Mol. Life Sci.

[36] Cohn RD, van Erp C, Habashi JP, Soleimani AA, Klein EC, Lisi MT, Gamradt M, Rhys CM, Holm TM, Loeys BL, Ramirez F, Judge DP, Ward CW, Dietz HC (2007). Angiotensin II type 1 receptor blockade attenuates TGF-beta-induced failure of muscle regeneration in multiple myopathic states. Nat. Med.

[37] Ng CM, Cheng A, Myers LA, Martinez-Murillo F, Jie C, Bedja D, Gabrielson KL, Hausladen JM, Mecham RP, Judge DP, Dietz HC (2004). TGF-beta-dependent pathogenesis of mitral valve prolapse in a mouse model of Marfan syndrome. J. Clin. Invest.

[38] Oshima A, Tanabe H, Yan T, Lowe GN, Glackin CA, Kudo A (2002). A novel mechanism for the regulation of osteoblast differentiation: transcription of periostin, a member of the fasciclin I family, is regulated by the bHLH transcription factor, twist. J. Cell Biochem.

[39] Yang J, Mani SA, Donaher JL, Ramaswamy S, Itzykson RA, Come C, Savagner P, Gitelman I, Richardson A, Weinberg RA (2004). Twist, a master regulator of morphogenesis, plays an essential role in tumor metastasis. Cell.

[40] Gronroos E, Terentiev AA, Punga T, Ericsson J (2004). YY1 inhibits the activation of the p53 tumor suppressor in response to genotoxic stress. Proc. Natl. Acad. Sci. USA.

[41] Gordon S, Akopyan G, Garban H, Bonavida B (2006). Transcription factor YY1: structure, function, and therapeutic implications in cancer biology. Oncogene.

[42] Wang CC, Chen JJ, Yang PC (2006). Multifunctional transcription factor YY1: a therapeutic target in human cancer?. Expert. Opin. Ther. Targets.

[43] Horiuchi K, Amizuka N, Takeshita S, Takamatsu H, Katsuura M, Ozawa H, Toyama Y, Bonewald LF, Kudo A (1999). Identification and characterization of a novel protein, periostin, with restricted expression to periosteum and periodontal ligament and increased expression by transforming growth factor beta. J. Bone Miner. Res.

[44] Woodruff PG, Boushey HA, Dolganov GM, Barker CS, Yang YH, Donnelly S, Ellwanger A, Sidhu SS, Dao-Pick TP, Pantoja C, Erle DJ, Yamamoto KR, Fahy JV (2007). Genome-wide profiling identifies epithelial cell genes associated with asthma and with treatment response to corticosteroids. Proc. Natl. Acad. Sci. USA.

[45] Shimazaki M, Nakamura K, Kii I, Kashima T, Amizuka N, Li M, Saito M, Fukuda K, Nishiyama T, Kitajima S, Saga Y, Fukayama M, Sata M, Kudo A (2008). Periostin is essential for cardiac healing after acute myocardial infarction. J. Exp. Med.

[46] Johnatty SE, Dyck JR, Michael LH, Olson EN, Abdellatif M (2000). Identification of genes regulated during mechanical load-induced cardiac hypertrophy. J. Mol. Cell Cardiol.

[47] Stanton LW, Garrard LJ, Damm D, Garrick BL, Lam A, Kapoun AM, Zheng Q, Protter AA, Schreiner GF, White RT (2000). Altered patterns of gene expression in response to myocardial infarction. Circ. Res.

[48] Wang D, Oparil S, Feng JA, Li P, Perry G, Chen LB, Dai M, John SW, Chen YF (2003). Effects of pressure overload on extracellular matrix expression in the heart of the atrial natriuretic peptide-null mouse. Hypertension.

[49] Chen YF, Feng JA, Li P, Xing D, Ambalavanan N, Oparil S (2006). Atrial natriuretic peptide-dependent modulation of hypoxia-induced pulmonary vascular remodeling. Life Sci.

[50] Lindner V, Wang Q, Conley BA, Friesel RE, Vary CP (2005). Vascular injury induces expression of periostin: implications for vascular cell differentiation and migration. Arterioscler. Thromb. Vasc. Biol.

[51] Hayashi N, Yoshimoto T, Izuhara K, Matsui K, Tanaka T, Nakanishi K (2007). T helper 1 cells stimulated with ovalbumin and IL-18 induce airway hyperresponsiveness and lung fibrosis by IFN-gamma and IL-13 production. Proc. Natl. Acad. Sci. USA.

[52] Roy S, Patel D, Khanna S, Gordillo GM, Biswas S, Fried-man A, Sen CK (2007). Transcriptome-wide analysis of blood vessels laser captured from human skin and chronic wound-edge tissue. Proc. Natl. Acad. Sci. USA.

[53] Spinale FG (2007). Myocardial matrix remodeling and the matrix metalloproteinases: influence on cardiac form and function. Physiol. Rev.

[54] Burlew BS, Weber KT (2002). Cardiac fibrosis as a cause of diastolic dysfunction. Herz.

[55] Lindsey ML, Mann DL, Entman ML, Spinale FG (2003). Extracellular matrix remodeling following myocardial injury. Ann. Med.

[56] Litvin J, Blagg A, Mu A, Matiwala S, Montgomery M, Ber-retta R, Houser S, Margulies K (2006). Periostin and periostin-like factor in the human heart: possible therapeutic targets. Cardiovasc. Pathol.

[57] Kühn B, del Monte F, Hajjar RJ, Chang YS, Lebeche D, Arab S, Keating MT (2007). Periostin induces proliferation of differentiated cardiomyocytes and promotes cardiac repair. Nat. Med.

[58] Sugiura T, Takamatsu H, Kudo A, Amann E (1995). Expression and characterization of murine osteoblast-specific factor 2 (OSF-2) in a baculovirus expression system. Protein Expr. Purif.

[59] Yan W, Shao R (2006). Transduction of a mesenchyme-specific gene periostin into 293T cells induces cell invasive activity through epithelial-mesenchymal transformation. J. Biol. Chem.

[60] Goerdt S, Bhardwaj R, Sorg C (1993). Inducible expression of MS-1 high-molecular-weight protein by endothelial cells of continuous origin and by dendritic cells/macrophages *in vivo* and *in vitro*. Am. J. Pathol.

[61] Politz O, Gratchev A, McCourt PA, Schledzewski K, Guillot P, Johansson S, Svineng G, Franke P, Kannicht C, Kzhyshkowska J, Longati P, Velten FW, Johansson S, Goerdt S (2002). Stabilin-1 and -2 constitute a novel family of fasciclin-like hyaluronan receptor homologues. Biochem. J.

[62] Kruzynska-Frejtag A, Wang J, Maeda M, Rogers R, Krug E, Hoffman S, Markwald RR, Conway SJ (2004). Periostin is expressed within the developing teeth at the sites of epithelial-mesenchymal interaction. Dev. Dyn.

[63] Horiuchi K, Amizuka N, Takeshita S, Takamatsu H, Katsuura M, Ozawa H, Toyama Y, Bonewald LF, Kudo A (1999). Identification and characterization of a novel protein, periostin, with restricted expression to periosteum and periodontal ligament and increased expression by transforming growth factor beta. J. Bone Miner. Res.

